# Dermatophytosis among Schoolchildren in Three Eco-climatic Zones of Mali

**DOI:** 10.1371/journal.pntd.0004675

**Published:** 2016-04-28

**Authors:** Oumar Coulibaly, Abdoulaye K. Kone, Safiatou Niaré-Doumbo, Siaka Goïta, Jean Gaudart, Abdoulaye A. Djimdé, Renaud Piarroux, Ogobara K. Doumbo, Mahamadou A. Thera, Stéphane Ranque

**Affiliations:** 1 IP-TPT UMR MD3, Aix-Marseille Université, Marseille, France; 2 Département d’Épidémiologie des Affections Parasitaires/Malaria Research and Training Center, Faculté de Médecine, Université des Sciences, des Techniques et Technologies de Bamako, Bamako, Mali; 3 SESSTIM, Institut National de la Santé et de la Recherche Médicale / Institut de Recherche pour le Développement / Aix-Marseille Université, Marseille, France; 4 Parasitologie & Mycologie, Hôpital de la Timone, Assistance Publique-Hôpitaux de Marseille, Marseille, France; University of Minnesota, UNITED STATES

## Abstract

**Background:**

Dermatophytosis, and particularly the subtype *tinea capitis*, is common among African children; however, the risk factors associated with this condition are poorly understood. To describe the epidemiology of dermatophytosis in distinct eco-climatic zones, three cross-sectional surveys were conducted in public primary schools located in the Sahelian, Sudanian and Sudano-Guinean eco-climatic zones in Mali.

**Principal Findings:**

Among 590 children (average age 9.7 years) the overall clinical prevalence of *tinea capitis* was 39.3%. *Tinea capitis* prevalence was 59.5% in the Sudano-Guinean zone, 41.6% in the Sudanian zone and 17% in the Sahelian eco-climatic zone. *Microsporum audouinii* was isolated primarily from large and/or microsporic lesions. *Trichophyton soudanense* was primarily isolated from trichophytic lesions. Based on the multivariate analysis, *tinea capitis* was independently associated with male gender (OR = 2.51, 95%CI [1.74–3.61], *P*<10^−4^) and residing in the Sudano-Guinean eco-climatic zone (OR = 7.45, 95%CI [4.63–11.99], *P*<10^−4^). Two anthropophilic dermatophytes species, *Trichophyton soudanense* and *Microsporum audouinii*, were the most frequent species associated with *tinea capitis* among primary schoolchildren in Mali.

**Conclusions:**

*Tinea capitis* risk increased with increasing climate humidity in this relatively homogenous schoolchild population in Mali, which suggests a significant role of climatic factors in the epidemiology of dermatophytosis.

## Author Summary

Dermatophytosis, and particularly the subtype *tinea capitis*, is common among African children; however, the risk factors associated with this condition are poorly understood. To describe the epidemiology of dermatophytosis in distinct eco-climatic zones, three cross-sectional surveys were conducted in public primary schools located in the Sahelian, Sudanian and Sudano-Guinean eco-climatic zones in Mali. Among 590 children (average age 9.7 years) the overall clinical prevalence of *tinea capitis* was 39.3%. *Tinea capitis* prevalence was 59.5% in the Sudano-Guinean zone, 41.6% in the Sudanian zone and 17% in the Sahelian eco-climatic zone. *Microsporum audouinii* was isolated primarily from large and/or microsporic lesions. *Trichophyton soudanense* was primarily isolated from trichophytic lesions. Based on the multivariate analysis, *tinea capitis* was independently associated with male gender (OR = 2.51, 95%CI [1.74–3.61], *P*<10^−4^) and residing in the Sudano-Guinean eco-climatic zone (OR = 7.45, 95%CI [4.63–11.99], *P*<10^−4^). Two anthropophilic dermatophytes species, *Trichophyton soudanense* and *Microsporum audouinii*, were the most frequent species associated with *tinea capitis* among primary schoolchildren in Mali. *Tinea capitis* risk increased with increasing climate humidity in this relatively homogenous schoolchild population, which strongly suggests that climatic factors play a significant role in the epidemiology of dermatophytosis.

## Introduction

Dermatophytosis represents one of the most common infectious diseases worldwide and causes serious chronic morbidity [1]. The condition is caused by dermatophytes, which are fungi that require keratin for growth. An increase in the incidence of such infections has been noted worldwide, especially in developing countries [2,3]. In particular, *tinea capitis* represents a major public health issue among children in developing countries. This dermatophytosis of the scalp and hair shafts is almost exclusively a childhood disease, and evidence suggests that it occurs more often in children of African or Caribbean origin [1]. Many factors including gender, age, urban/rural environment, socio-economic level and cultural habits have been shown to significantly impact the development of dermatophytosis worldwide, especially throughout the African continent [4–9]. It has been hypothesized that climate also plays an important role in the heterogeneity of dermatophytosis epidemiology in Africa [10]. Many studies have described dermatophytosis epidemiology in various geographical settings throughout the African continent. However, heterogeneous study design limits the assessment of numerous potential confounding factors and the striking differences observed cannot reliably be attributed to variations in climate. In fact, no study has been established to specifically address the impact of climate on dermatophytosis presentation. In Mali, the climate ranges from subtropical in the south to arid in the north. Therefore, the current study aimed to assess the prevalence, risk factors and etiological agents of *tinea capitis*, the most frequent dermatophytosis subtype, among primary schoolchildren in three eco-climatic zones in Mali.

## Methods

### Study areas and population

**Fig 1:**
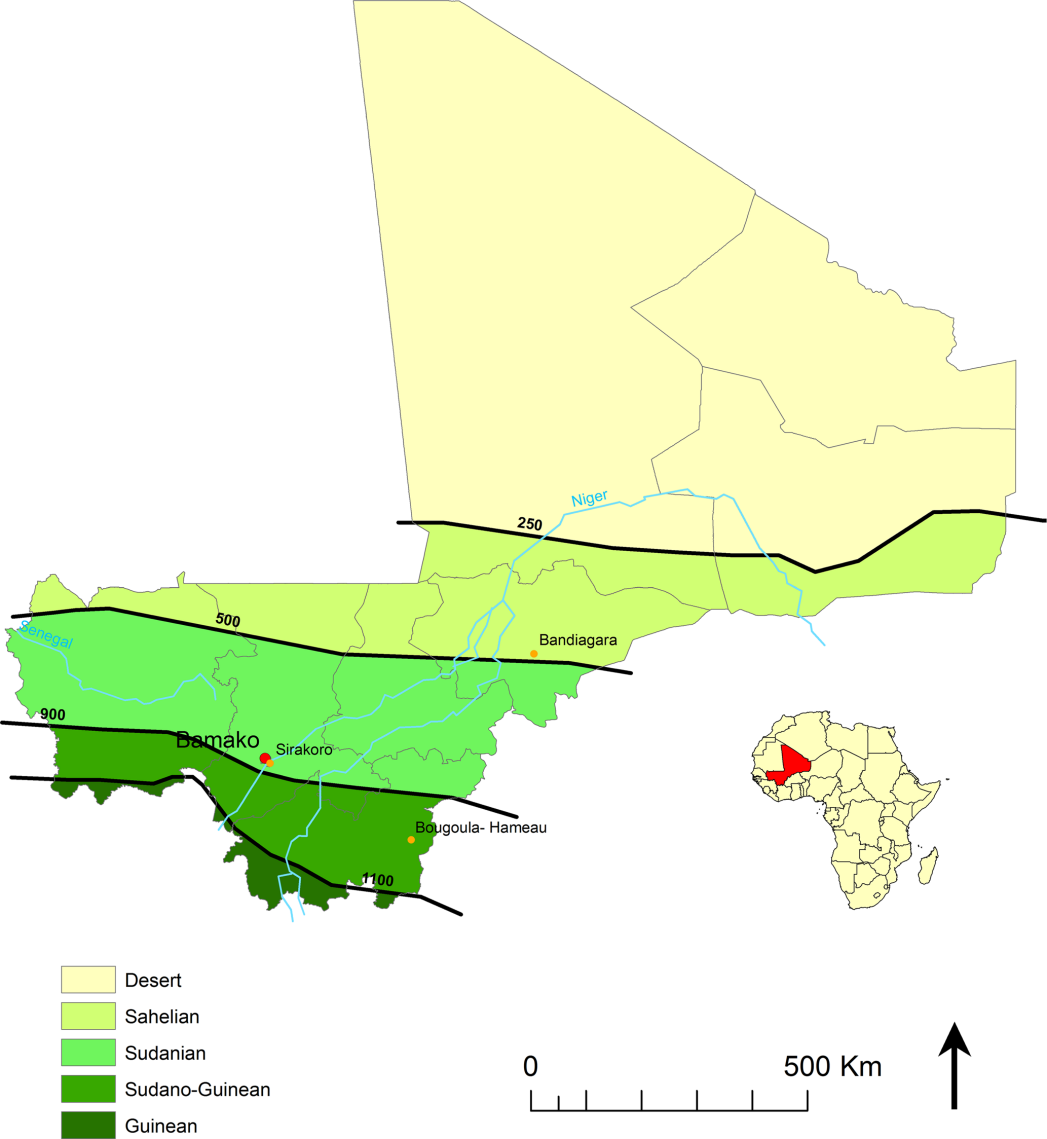
Study sites. Location of three study sites and the isohyets (mm/year) separating the Sahelian, Sudanian and Sudano-Guinean eco-climatic zones in Mali.

Three cross-sectional surveys were carried out during the dry season, in December 2009, December 2010 and February 2012, in three public primary schools in (i) Sirakoro-Meguetana, a semi-urban community located in the suburbs of Bamako, the capital of Mali, in the Sudanian eco-climatic zone; (ii) Bandiagara, a urban community in the Sahelian eco-climatic zone and (iii) Bougoula-Hameau, a semi-urban community located in the suburbs of Sikasso in the Sudano-Guinean eco-climatic zone. The study sites and eco-climatic zones previously defined [11] are shown on the map in Fig 1.

The characteristics of each eco-climatic zone, from north to south, are detailed below. The Sahelian zone is arid with annual rainfall levels between 250 to 550 mm. In Sévaré, located 55 km from Bandiagara, the average rainfall levels range from 0 mm in January to 161 mm in August, with total annual precipitation of approximately 485 mm. The mean temperatures in Sévaré range from 23.2°C in January to 33.0°C in May, with an average annual temperature of 28.3°C. The Sudanian zone is semi-arid to sub-humid with annual rainfall ranging between 550 and 1100 mm. In Bamako, the average rainfall ranges from 0 mm in January to 291 mm in August, with total annual precipitation of approximately 955 mm. The mean temperatures in Bamako range from 25.0°C in December to 31.5°C. The Sudano-Guinean zone is sub-humid with average annual rainfall greater than 1100 mm. In Sikasso, the average rainfall levels range from 1 mm in January to 298 mm in August, with total annual precipitation of approximately 1125 mm. The mean temperatures in Sikasso range from 23.8°C in January to 30.2°C in April, with an average annual temperature of 28.3°C.

### Study participants

Pupils, aged 6 to 15 years, were randomly selected in each primary school using a block randomization design adjusted on the number of pupils in each classroom. Oral informed consent was obtained from the children and their parents or guardians. The exclusion criterion was a history of antifungal treatment (oral or topical, conventional or traditional) within two weeks. Medical history and information concerning exposure to potential dermatophytosis risk factors were recorded, including contact with animals and specific hair grooming habits, and a complete physical examination of the skin and appendages, including fingernails and hair, was performed on all children by one of the investigators. The data were recorded on a standardized clinical report form.

### Ethical issues and biological samples collection

The study protocol was reviewed and approved by the Faculty of Medicine’s Institutional Review Board at the University of Bamako, Mali. The study protocol was also approved by the Local Education Authorities at each study site. Parent or guardian provided written informed consent on behalf of the participating children.

**Fig 2:**
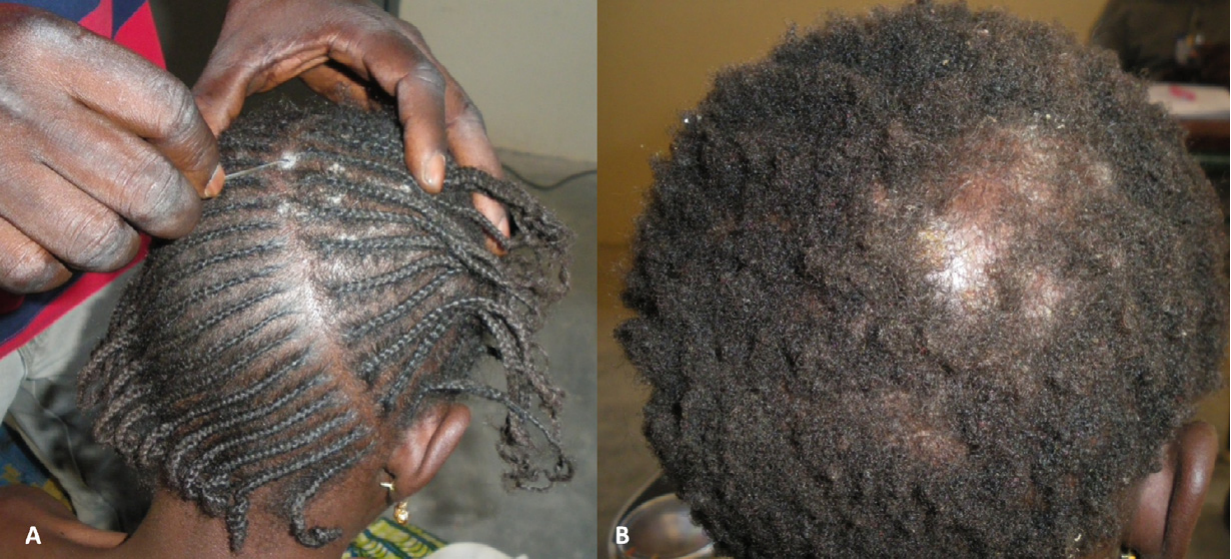
*Tinea capitis* clinical presentation. (A) Trichophytic presentation with diffuse relatively small scalp lesions, mainly involving *Trichophyton soudanense*. (B) Microsporic presentation with relatively large and scarce lesions, mainly involving *Microsporum audouinii*.

Samples were collected from each lesion that was compatible with dermatophytosis. Skin samples were isolated from the peripheral erythematous border of the lesion. Scalp lesions were collected by scraping the area with a sterile curette, and broken and lusterless hairs were selected and plucked using sterile tweezers (Fig 2A). One portion of the each sample was used for direct examination via microscopy, while the second portion was inoculated directly onto Sabouraud Dextrose Agar (SDA) (bioMérieux, Marcy l’Etoile, France) with antibiotics and cycloheximide for mycological examination. In the Sahelian and Sudano-Guinean zones, an additional sample was collected directly from the same lesion via sterile gauze. The samples inoculated on SDA were incubated at room temperature before being transferred to the Parasitology-Mycology Laboratory at the University Hospital of Marseilles, where they were incubated at 27°C for a of 4-6-week period. The sterile gauze samples collected in the Sahelian and Sudano-Guinean zones were stored at ambient temperature in individually sealed plastic bags before being inoculated on SDA agar (BioMérieux) and subsequently incubated for 4–6 weeks at 27°C at the Parasitology-Mycology Laboratory in Marseilles. Dermatophyte colonies were identified based on examination of the macro- and micro-morphological features of the fungus, and those with atypical morphological features were further identified via rDNA internal transcribed spacer 2 (ITS2) sequence analysis as previously described [12].

### Statistical analysis

A sample size of 200 children in each study site was calculated to estimate a 12% dermatophytosis prevalence rate with a 4.5% precision at α = 5%. The data were analyzed using SAS 9.2 for Windows (SAS Institute Inc., Cary, NC, USA). Continuous variables were expressed as the mean (SD), while categorical variables were expressed as proportions and percentages. Continuous variables were compared using ANOVA. Categorical variables were compared using the Chi square or Fisher's exact tests as required. All statistical tests were two-sided with a *P*<0.05 significance level. Univariate and multivariate unconditional logistic regression analyses were performed to estimate odds ratios (ORs) with a 95% confidence interval (CI). All covariates with a *P*<0.20 significance level in the univariate analysis were included in the multivariate logistic regression model. A stepwise selection was performed to retain the most parsimonious model including the covariates that displayed an independent statistically significant (*P*<0.05) effect on *tinea capitis* risk.

## Results

### Study population

Of the 590 randomly selected schoolchildren, 286 males and 304 females participated in this study, including 190 from Sirakoro-Meguetana, 200 from Bandiagara and 200 from Sikasso (Fig 1). Although the age distribution of the participating children in each eco-climatic zone differed significantly (*P* = 0.001), the mean age of each zone, which ranged from 9.3 to 10.2 years, was quite similar (Table 1). The sex ratio of the participating schoolchildren in each eco-climatic zone did not significantly differ (*P =* 0.151). As expected, the ethnic group distribution of each eco-climatic zone differed significantly (*P*<0.001) (Table 1).

**Table 1:** Demographic, clinical and mycological features of the 590 children that participated in the study grouped by eco-climatic zones in Mali.

			Eco-climatic zones						
Demographic data	Total		Sahelian		Sudanian		Sudano-Guinean		
**All participants**	n = 590	%	n = 200	%	n = 190	%	n = 200	%	***P* values**
**Gender**	590	100	200	100	190	100	200	100	0.151
Female	304	51.5	104	52	107	56.3	93	46.5	
Male	286	48.5	96	48	83	43.7	107	53.5	
**Age** Mean (SD)	570	9.7 (2.38)	192	9.6 (2.3)	185	9.3 (2.4)	193	10.2 (2.3)	0.001
**Ethnic group**	547	92.7	195	97.5	189	99.5	163	81.5	<0.001
Dogon	219	40	134	68.7	85	45	0	0	
Bambara	60	11	14	7.2	31	16.4	15	9.2	
Fulani	52	9.5	24	12.3	24	12.7	4	2.4	
Senufo	134	24.5	0	0	0	0	134	82.2	
Other	82	15	23	11.8	49	25.9	10	6.2	
**Tinea capitis population**	**n = 232**	**%**	**n = 34**	**%**	**n = 79**	**%**	**n = 119**	**%**	***P* value**
**Age** Mean (SD)	232	9.8 (2.4)	34	9.6 (2.4)	79	9.4 (2.5)	119	10.1 (2.3)	0.132
**Sex group**	232	100	34	100	79	100	119	100	0.020
Female	90	38.8	9	26.5	40	50.6	41	34.4	-
Male	142	61.2	25	73.5	39	49.4	78	65.6	-
**Clinical features**									
Inflammatory	4	1.7	0	0	2	2.5	2	1.7	0.637
Suppurative	3	1.3	1	2.9	0	0	2	1.7	0.387
Diffuse	116	50.0	9	26.5	41	51.9	66	55.5	0.011
Size >2 cm	100	43.1	12	35.3	17	21.5	71	59.7	<0.001
Number >2	169	72.8	27	79.4	79	100	63	52.9	<0.001
Trichophytosis	176	75.9	22	64.7	62	78.5	92	77.3	0.254
Microsporosis	54	23.3	12	35.3	15	19	27	22.7	0.166
*Hair shaft invasion*									
Endothrix	50	20.6	11	32.3	14	17.7	25	21	0.205
Endo-ectothrix	55	23.7	10	29.4	12	15.2	33	27.7	0.089
**Dermatophytes**									
**Culture**	232	100	34	100	79	100	119	100	-
Positive	189	81.5	34	100	36	45.6	119	100	-
Negative	19	8.2	0	0.0	19	24.0	0	0.0	-
**Species**	213	100	34	100	60	100	119	100	-
*M*. *audouinii*	69	32.4	12	35.3	10	16.7	47	39.5	-
*T*. *soudanense*	78	36.6	22	64.7	19	31.7	37	31.1	-
*T*. *violaceum*	7	3.3	0	0.0	0	0.0	7	5.9	-
*T*. *mentagrophytes*	4	1.9	0	0.0	4	6.6	0	0.0	-
*T*. *s*. + *T*. *m*.	3	1.4	0	0.0	3	5.0	0	0.0	-
*T*. *s*. + *M*. *a*.	28	13.2	0	0.0	0	0.0	28	23.5	-
NDF^1^	24	11.3	0	0.0	24	40.0	0	0.0	-

1 NDF: Non-dermatophyte filamentous fungi

### Dermatophytosis prevalence

As detailed in Table 1, 312 children presented with clinical dermatophytosis lesions, thereby yielding an overall dermatophytosis prevalence of 52.9% (95% CI [48.7–57.0]) among the entire study population. The most frequent (232/590) clinical presentation was *tinea capitis* with 39.3% 95% CI [35.4–43.4] prevalence. Therefore, *tinea capitis* was separately detailed in the study, and all other clinical presentations of dermatophytosis were categorized as non-*tinea capitis*, which was found in 80 (13.6%, 95% CI [10.9–16.6]) of the participating children. Among the 80 non-*tinea capitis* dermatophytosis cases, the most common clinical presentation was *tinea corporis* (81.3%), followed by *tinea cruris* (8.7%), *tinea pedis* (7.5%) and *tinea unguium* (2.5%), irrespective of the geographic area.

### *Tinea capitis* prevalence and clinical presentation

Overall, 39.3% (95% CI [35.4–43.4]) of the schoolchildren presented with clinical *tinea capitis* lesions. The prevalence of *tinea capitis* significantly (P<0.001) differed depending on the geographic area, with prevalence rates of 17.0% (95% CI [12.1–22.9]), 41.6% (95% CI [34.5–48.9]) and 59.5% (95% CI [52.4–66.4]) recorded in the Sahelian, Sudanian and Sudano-Guinean eco-climatic zones, respectively. The characteristics of the 232 schoolchildren presenting with clinical *tinea capitis* lesions are detailed in Table 1. The *tinea capitis* lesions were described as diffuse primarily in the Sudano-Guinean (55.5%) and Sudanian (51.9%) zones, while diffuse lesions were observed only in 26.5% of cases in the Sahelian zone (P = 0.012). Interestingly, this individual marker of infection intensity correlated with prevalence. Inflammatory and suppurative forms of *tinea capitis* were rarely observed. *Tinea capitis* presentation in the Sudano-Guinean zone was characterized by the predominance of microsporosis (Fig 2B), *i*.*e*. large lesions (>2 cm) involving *Microsporum audouinii*, in contrast to the Sahelian and Sudanian zones, which were characterized by the predominance of trichophytosis (Fig 2A), *i*.*e*. multiple (n>2) lesions involving *Trichophyton soudanense* (Table 1). As expected, the majority of microsporosis lesions (64.5%) and a particularly high proportion (80.3%) of large (>2 cm) *tinea capitis* lesions, displayed a typical green fluorescence upon Wood’s lamp examination, while most trichophytosis lesions (95.5%) did not display this feature. Of note, 30.3% of the diffuse scalp lesions tested positive upon Wood’s lamp examination.

### Non-*tinea capitis* dermatophytosis

Eighty children (13.6%) presented with non-*tinea capitis* clinical dermatophytosis (Table 2), of which *tinea corporis* was the most frequent (81.3%) clinical presentation. The prevalence of *tinea corporis* varied depending on the eco-climatic zone, ranging from 88.4% in the Sudanian to 71.4% in the Sudano-Guinean zone. Overall, six children (7.5%) presented with athlete’s foot, of which four (14.3%) originated from the Sudano-Guinean zone and two (4.7%) were from the Sudanian zone.

**Table 2:** Clinical presentation and mycological features of non-*tinea capitis* dermatophytosis according to eco-climatic zones in Mali.

			Eco-climatic zones					
	Total		Sahelian		Sudanian		Sudano-Guinean		
	n = 80	%	n = 9	%	n = 43	%	n = 28	%	
**Clinical features**									
*Tinea corporis*	65	81.3	7	77.8	38	88.4	20	71.4	
*Tinea cruris*	7	8.7	2	22.2	1	2.3	4	14.3	
*Tinea pedis*	6	7.5	0	0.0	2	4.7	4	14.3	
*Tinea unguium*	2	2.5	0	0.0	2	4.6	0	0.0	
**Direct examination features**									
Positive	3	3.8	0	0.0	3	7.0	0	0.0	
Negative	77	96.2	9	100	41	93.0	28	100	
**Dermatophytes culture**									
Positive	58	72.5	9	100	27	62.8	22	78.6	
Negative	10	12.5	0	0.0	4	9.3	6	21.4	
NDF*	12	15.0	0	0.0	12	27.9	0	0.0	
**Fungal species isolated**									
*T*. *soudanense*	51	63.8	9	100	20	40.8	22	100	
*T*. *mentagrophytes*	4	5.0	0	0.0	4	8.2	0	0.0	
*T*. *s + T*. *m*^1^	3	3.8	0	0.0	3	6.1	0	0.0	
NDF^2^	22	27.4	0	0.0	22	44.9	0	0.0	

1. *T*. *s* + *T*. *m*: *T*. *soudanense* and *T*. *mentagrophytes*;

2. NDF: Non-dermatophyte filamentous fungi

### Mycological findings

The majority of *tinea capitis* lesions (81.5%) tested positive upon direct microscopic examination. The distribution of endothrix or endo-ectothrix parasitism type did not significantly differ between the geographic areas (Table 1). In contrast, only 3.8% of the non-*tinea capitis* dermatophytosis samples tested positive upon direct examination (Table 3).

**Table 3:** Clinical, direct microscopic and Wood’s lamp examination features of *tinea capitis* lesions according to the identified dermatophyte species and non-dermatophyte filamentous fungus.

Species^1^	*M*. *aud*.		*T*. *soud*.		*T*. *menta*.		*T*. *viol*.		*T*. *s*. *+ T*. *m*.		*T*. *s*. *+ M*. *a*.		NDF		
(n = 213)	n = 69	%	n = 78	%	n = 4	%	n = 7	%	n = 3	%	n = 28	%	n = 24	%	*P* values
**Clinical features**															
Inflammatory (n = 4)	0	0.0	0	0.0	2	50.0	2	28.6	0	0.0	0	0.0	0	0.0	<0.001
Suppurative (n = 3)	0	0.0	1	1.3	0	0.0	2	28.6	0	0.0	0	0.0	0	0.0	<0.001
Diffuse (n = 105)	17	24.6	44	56.4	4	100	5	71.4	3	100	18	34.3	14	58.3	<0.001
Size >2 cm (n = 97)	52	75.4	16	20.5	1	25	5	71.4	1	33.3	21	75	1	4.2	<0.001
Number >2	38	57.1	55	70.5	4	100	4	57.0	3	100	22	78.6	24	100	0.001
Trichophytosis (n = 160)	22	31.9	75	96.2	3	75	7	100	3	100	28	100	22	91.7	<0.001
Microsporosis (n = 51)	47	68.1	1	1.3	1	25	0	0.0	0	0.0	1	3.6	1	4.2	<0.001
**Direct examination features**															
Positive (n = 99)	48	69.6	27	34.6	0	0.0	3	42.9	2	66.7	14	50.0	2	20.8	0.001
Endothrix (n = 47)	2	2.9	27	34.6	0	0.0	3	42.9	2	66.7	9	32.1	4	16.6	<0.001
Endoectothrix (n = 53)	46	66.7	0	0.0	0	0.0	0	0.0	0	0.0	6	21.4	1	4.2	<0.001
**Wood’s lamp examination features**															
Wood + (n = 72)	59	85.5	3	3.8	0	0.0	0	0.0	0	0.0	9	32	1	4.2	<0.001
Wood—(n = 141)	10	14.5	75	96.2	4	100	7	100	3	100	19	68.0	23	95.8	

1. *M*. *aud*.: *Microsporum audouinii*; *T*. *soud*.: *Trichophyton soudanense*; *T*. *menta*.: *Trichophyton mentagrophytes*; *T*. *viol*.: *Trichophyton violaceum*; *T*. *s*. + *T*. *m*.: *T*. *soudanense* and *T*. *mentagrophytes*; *T*. *s*. + *M*. *a*.: *T*. *soudanense* and *M*. *audouinii*; NDF: non-dermatophyte filamentous fungi.

#### Dermatophyte culture

Of 312 samples collected from all clinical dermatophytosis lesions, 247 mycological cultures tested positive for dermatophyte fungus, thereby yielding an overall dermatophyte culture sensitivity of 79.2% (95%CI [74.2–83.5]). Dermatophyte cultures sensitivity for both *tinea capitis* and non-*tinea capitis* diagnosis were lowest in the first study site located in the Sudan eco-climatic zone (Tables 1 and 2).

#### Dermatophytes isolated from *tinea capitis*

Mycological culture analysis yielded dermatophytes in 189 of the 232 *tinea capitis* samples collected, thereby yielding a global prevalence of 32% (189/590) mycologically-confirmed cases. A positive mycological cultures rate was 100% in the Sahelian and Sudano-Guinean zones but 45.6% (36/79) in the Sudanian zone, where 24% of mycological cultures were negative and 30.4% were contaminated with non-dermatophytes filamentous fungi. Overall, we recovered 24 non-dermatophyte filamentous fungi and 189 dermatophyte isolates of four dermatophyte species, namely *Trichophyton soudanense*, *T*. *mentagrophytes*, *T*. *violaceum* and *Microsporum audouinii* (Table 3). In 14.5% of the positive cultures, two dermatophyte species were co-isolated, namely *T*. *soudanense* together with either *T*. *mentagrophytes* or *M*. *audouinii*. The *tinea capitis* clinical features associated with each dermatophyte species, or combination of species, are detailed in Table 3. *M*. *audouinii* was isolated primarily from large (>2 cm) lesions (75.4%) and/or microsporic lesions (68.1%). *T*. *soudanense* was primarily isolated from trichophytic lesions (96.2%). The combination of both *T*. *soudanense* and *M*. *audouinii* was found only in trichophytic lesions. The Wood’s lamp examination tested positive in 85.5% (*P* < .0001) of *tinea capitis* cases involving *M*. *audouinii* (Table 3). A predominance of anthropophilic species was observed in the three eco-climatic zones (Table 3). In the Sahelian zone, a clear predominance of *T*. *soudanense* was observed, which displayed a prevalence of 64.7%, 31.7% and 31.1% in the Sahelian, Sudanian and Sudano-Guinean eco-climatic zones, respectively (*P*<0.001). *M*. *audouinii* was the most common etiological agent (39.5%) in Bougoula-Hameau. Of note, *T*. *violaceum* was only isolated in the Sudano-Guinean eco-climatic zone, while *T*. *mentagrophytes* was found only in the Sudanian zone.

### Dermatophytes isolated from other dermatophytosis samples

A dermatophyte was isolated in 58 of the 80 samples collected from children with *tinea corporis*, *tinea cruris*, athlete foot or onychomycosis, thereby yielding a global prevalence of 9.8% (95%CI [7.6–12.5]) of mycologically confirmed non-*tinea capitis* dermatophytosis cases (Table 3). Two dermatophyte species were predominant, namely *T*. *soudanense* (63.8%) and *T*. *mentagrophytes* (5%). The combination of both *T*. *soudanense* and *T*. *mentagrophytes* was found in 3.8% of positive cultures. *M*. *audouinii* was not isolated from non-*tinea capitis* lesions in this study. Twenty-two (27.4%) non-dermatophyte filamentous fungi were recovered, primarily in the Sudanian eco-climatic zone (Table 3).

### *Tinea capitis* risk factors

The global and gender-specific distributions of the assessed *tinea capitis* risk factors are tabulated in Table 1. The distribution of the potential risk factors and habits associated with *tinea capitis* among schoolchildren according to their *tinea capitis* status is shown in Suppl Table 1. In the univariate analysis, several hairdressing habits were found significantly associated with *tinea capitis*; however, the associations were non-significant when gender was considered, which acted as a notable confounding factor. Proximity to cattle was associated with a significant decrease in *tinea capitis* risk among females but not among males. In contrast, the presence of a dog in the household was associated with increased *tinea capitis* risk among males but not among females. None of these risk factors were found statistically significant in the multivariate analysis. The effects of the most significant *tinea capitis* risk factors are detailed in Table 4. In the univariate analysis, *tinea capitis* was significantly associated with male gender (OR = 7.85, 95%CI [5.22–11.81], *P*<0.001) and residing in Bougoula-Hameau (OR = 7.17, 95%CI [4.51–11.4], *P*<0.001). Notably, age had no significant effect on this study population. In the multivariate analysis, both male gender (OR = 2.51, 95%CI [1.74–3.61], *P*<0.001) and residing in the Sudano-Guinean eco-climatic zone (OR = 7.45, 95%CI [4.63–11.99], *P*<0.001) were statistically significant independent *tinea capitis* risk factors (Table 4).

**Table 4:** Univariate and multivariate unconditional logistic regression analyses of the *tinea capitis* risk factors identified in this population.

	Univariate analysis			Multivariate analysis		
	OR	95%CI	*P* value	OR	95%CI	*P* value
**Age group (years)**						
< 8	0.73	0.43–1.24	0.448	-	-	-
[8–10]	0.73	0.41–1.29	0.489	-	-	-
[10–12]	0.81	0.46–1.42	0.984	-	-	-
≥ 12	1	-	-	-	-	-
**Male gender**	2.34	1.67–3.29	<0.001	2.51	1.74–3.61	<0.001
**Eco-climatic zones**						
Sahelian	1	-	-	1	-	-
Sudanian	3.48	2.18–5.55	0.168	3.81	2.36–6.17	0.085
Sudano-Guinean	7.17	4.51–11.4	<0.001	7.45	4.63–11.99	<0.001

## Discussion

Overall, our study highlights three major findings: a dramatic disparity in *tinea capitis* epidemiology between distinct eco-climatic zones within Mali, evidence of an increased *tinea capitis* risk among male children and the dermatophyte species distribution. The dermatophytosis prevalence variations observed in the current study might be associated with varied exposure of the surveyed schoolchildren in each area to several risk factors, including eco-climatic, socio-economic factors and genetic or ethno-cultural elements. Notably, with the exception of ethno-cultural characteristics, the schoolchildren surveyed in each area were homogenous, especially in regards to age, and each survey was performed at the same period during the dry season. Therefore, the observed geographical differences in *tinea capitis* epidemiology may be attributed to variations in the local environment, and more specifically eco-climatic differences. Indeed, the highest prevalence of dermatophytosis was recorded in Bougoula-Hameau (59.5%), where the Sudano-Guinean climate is characterized by relatively higher levels of humidity; followed by Sirakoro-Meguetana (41.6%), where the Sudanian climate is characterized by intermediate humidity levels; and Bandiagara (17%), where the Sahelian climate is characterized by relatively lower humidity levels. This association trend of *tinea capitis* risk that increases with the humidity level of the climate correlates with data from Togo, where *tinea capitis* prevalence rates were 11% in a dry region in the North of the country and 20% in a humid area in the South of the country [13]. These geographical discrepancies might be associated with hot and humid climates, which favor the growth and spread of fungi and may predispose populations to various skin diseases including *tinea capitis* [14].

*Tinea capitis* primarily affects children in developing countries, while *tinea pedis* and *tinea unguium* pose the greatest burden to adults and the elderly in developed countries [15]. The high prevalence dermatophytosis rate (52.9%) found in this study is similar with those reported in many studies concerning African schoolchildren [16–19]. In correlation with our findings, *tinea capitis* was also the most frequent dermatophytosis presentation among children [4–6,20]. Its 39.3% prevalence rate was similar to those observed among outpatients attending the dermatology consultation at the Marchoux Dermatology Institute in Bamako (38.5%) [21] and higher than those reported in previous surveys of schoolchildren in Bamako (7% and 12.5%) [22,23].

In agreement with the majority of African studies, our findings highlight male gender as a significant *tinea capitis* risk factor among schoolchildren [6,7,16,19,22–24]. For example, in Mali, the prevalence of *tinea capitis* was 4.4% and 2.1% (*P*< 10^−6^) in schoolboys and schoolgirls, respectively [22]. Meanwhile, the prevalence was 3 and 5 times higher in boys than in girls in Abidjan (Côte d’Ivoire) and Central Nigeria, respectively [6,7].

Many potential risk factors for *tinea capitis* have been proposed [25]. Although our study did not address genetic susceptibility to dermatophytosis, we considered ethno-cultural risk factors, which are likely to play a significant role in dermatophytosis epidemiology. It has been reported that dermatophytosis prevalence is influenced by the hairdressing mode, extracurricular activity and cultural habits, rather than population density [4,6]. In the present study population, the following factors were associated with *tinea capitis* in the univariate but not in the multivariate analysis: contact with dogs (*P* = 0.002), public hairdressing practices (P = 0.022), home hairdressing practices (*P*<0.001), traditional braiding practices (*P*<0.001) and head shaving practices (*P*<0.001). These differences between crude and adjusted risk estimates in this study are caused by multicollinearity, nested effects (i.e. contact with animals and contact with dogs …) or non-independence with quasi-complete separation of data points (i.e. type of hairdressing mode (braiding or head shaving) according to the gender) among the predictors in the multivariate analysis. In Egypt, El-Khalawany et al. [25] have shown that contact with animals was a common predisposing factor for *tinea capitis* in rural areas, whereas transmission from other family members was more common among individuals residing in urban areas. However, increased dermatophyte risk due to contact with dogs was unexpected in our study, as we did not isolate *Microsporum canis*, the dermatophyte species usually associated with canines. Further studies are required to assess whether dogs might be involved in anthropophilic dermatophyte species transmission.

One limitation of the study is the relatively high false-negative dermatophyte culture rate in the first study compared with the two other study sites, which is associated with the subsequent introduction of sterile gauze in the sampling procedure. This limitation was taken into account by applying a clinical definition of *tinea capitis* in the risk factor study. We can reasonably presume that the false-negative culture results occurred at random in Sirakoro-Meguetana and thus did not alter the dermatophyte species distribution evaluation. As observed in other studies [5,7] the most common *Trichophyton* species was *T*. *soudanense* (36.6%), which was the most common species associated with *tinea capitis* in the Sahelian climate zone (64.7%). *T*. *soudanense* is one of the most common clinical dermatophyte species in West and Central Africa, where infections of this anthropophilic species are spread via direct contact between people. Two other species of the *Trichophyton* genus were isolated in this study: *T*. *mentagrophytes* (exclusively in the Sudanian zone in 6.6% of cases) and *T*. *violaceum* (only in the Sudano-Guinean zone in 5.9% of cases). The anthropophilic species *T*. *violaceum* has been shown associated with *tinea capitis* in Conakry-Guinea, where a 56.7% prevalence of this species was reported [26]. However, in Côte d’Ivoire, a low prevalence (2.3%) of this species has also been associated with *tinea capitis* [5]. It should be noted that *T*. *soudanense* and *T*. *violaceum* are phylogenetically very similar and that some experts consider these two taxa to be synonyms [27]. *M*. *audouinii* was the only species of the *Microsporum* genus isolated in this study. It was the second most common species following *T*. *soudanense*, which correlates with previous studies of schoolchildren in Bamako [22,23].

In conclusion, *tinea capitis* was diagnosed in 39.3% of a representative population of Malian schoolchildren. Two anthropophilic dermatophyte species, *T*. *soudanense* and *M*. *audouinii* were isolated in the majority of cases. Male gender and residing in the tropical North Guinea climatic zone of Mali were identified as independent *tinea capitis* risk factors. *Tinea capitis* risk increased with increasing humidity among the relatively homogenous populations located in distinct climatic geographic areas, thereby indicating that climatic factors may play a significant role in dermatophytosis epidemiology. Further epidemiological studies are required to elucidate the respective role that climatic and ethno-cultural factors play in dermatophytosis distribution.

## Supporting Information

S1 Checklist STROBE Checklist. (DOCX)

S1 Table Common hairdressing habits and domestic animal contact among the 590 children, according to gender and *tinea capitis* status. (DOCX)
