# Supplementary material for: Dermatophytosis among Schoolchildren in Three Eco-climatic Zones of Mali
Source: PLoS Negl Trop Dis. 2016 Apr 28;10(4):e0004675. doi: 10.1371/journal.pntd.0004675 (PMC4849727; doi:10.1371/journal.pntd.0004675)
Supplement: S1 Checklist — (DOCX) [file pntd.0004675.s001.docx]

STROBE Statement—checklist of items that should be included in reports of observational studies

|  | Item No. | Recommendation | Page  No. | Relevant text from manuscript |
| --- | --- | --- | --- | --- |
| **Title and abstract** | 1 | (*a*) Indicate the study’s design with a commonly used term in the title or the abstract | 2 | three cross-sectional surveys |
|  |  | (*b*) Provide in the abstract an informative and balanced summary of what was done and what was found | 2 |  |
| Introduction | | | |  |
| Background/rationale | 2 | Explain the scientific background and rationale for the investigation being reported | 4 | no study has been established to specifically address the impact of climate on dermatophytosis presentation |
| Objectives | 3 | State specific objectives, including any prespecified hypotheses | 4 | the current study aimed to assess the prevalence, risk factors and etiological agents of *tinea capitis* among primary schoolchildren in three eco-climatic zones in Mali |
| Methods | | | |  |
| Study design | 4 | Present key elements of study design early in the paper | 5 | Three cross-sectional surveys |
| Setting | 5 | Describe the setting, locations, and relevant dates, including periods of recruitment, exposure, follow-up, and data collection | 5 | Study areas and population section |
| Participants | 6 | (*a*) *Cross-sectional study*—Give the eligibility criteria, and the sources and methods of selection of participants | 6 | Pupils, aged 6 to 15 years, were randomly selected in each primary school using a block randomization design adjusted on the number of pupils in each classroom |
|  |  | (*b*) *Cohort study*—For matched studies, give matching criteria and number of exposed and unexposed  *Case-control study*—For matched studies, give matching criteria and the number of controls per case | NR | NR |
| Variables | 7 | Clearly define all outcomes, exposures, predictors, potential confounders, and effect modifiers. Give diagnostic criteria, if applicable | 6 | Medical history and information concerning exposure to potential dermatophytosis risk factors were recorded, including contact with animals and specific hair grooming habits, and a complete physical examination of the skin and appendages, including fingernails and hair, was performed on all children by one of the investigators. The data were recorded on a standardized clinical report form. |
| Data sources/ measurement | 8* | For each variable of interest, give sources of data and details of methods of assessment (measurement). Describe comparability of assessment methods if there is more than one group | *6* | *Se before* |
| Bias | 9 | Describe any efforts to address potential sources of bias | 6 | Pupils, aged 6 to 15 years, were randomly selected in each primary school using a block randomization design adjusted on the number of pupils in each classroom |
| Study size | 10 | Explain how the study size was arrived at | 7 | A sample size of 200 children in each study site was calculated to estimate a 12% dermatophytosis prevalence rate with a 4.5% precision at alpha=5%. |

Continued on next page

| Quantitative variables | 11 | Explain how quantitative variables were handled in the analyses. If applicable, describe which groupings were chosen and why | 7 | Continuous variables were expressed as the mean (SD), … were compared using ANOVA. |
| --- | --- | --- | --- | --- |
| Statistical methods | 12 | (*a*) Describe all statistical methods, including those used to control for confounding | 7 | Univariate and multivariate unconditional logistic regression analyses were performed to estimate odds ratios (ORs) with a 95% confidence interval (CI). All covariates with a *P*<0.20 significance level in the univariate analysis were included in the multivariate logistic regression model. A stepwise selection was performed to retain the most parsimonious model including the covariates that displayed an independent statistically significant (*P*<0.05) effect on *tinea capitis* risk. |
|  |  | (*b*) Describe any methods used to examine subgroups and interactions | NR | NR |
|  |  | (*c*) Explain how missing data were addressed | NR | Missing data were addressed as missing (there were not much) |
|  |  | (*d*) *Cross-sectional study*—If applicable, describe analytical methods taking account of sampling strategy | NR | The sampling was random. |
|  |  | (*e*) Describe any sensitivity analyses | ND | Not done. Yet the stepwise selection process that allows dropping or adding variables at the various steps, is somewhat similar to sensitivity analysis |
| Results | | | | |
| Participants | 13* | (a) Report numbers of individuals at each stage of study—eg numbers potentially eligible, examined for eligibility, confirmed eligible, included in the study, completing follow-up, and analysed | 8 | Of the 590 randomly selected schoolchildren, 286 males and 304 females participated in this study, including 190 from Sirakoro-Meguetana, 200 from Bandiagara and 200 from Sikasso |
|  |  | (b) Give reasons for non-participation at each stage | ND | ND |
|  |  | (c) Consider use of a flow diagram | NR | Not relevant |
| Descriptive data | 14* | (a) Give characteristics of study participants (eg demographic, clinical, social) and information on exposures and potential confounders | 9 | Table 1 |
|  |  | (b) Indicate number of participants with missing data for each variable of interest | 9-10 | Table 1 |
|  |  | (c) *Cohort study*—Summarise follow-up time (eg, average and total amount) | NR | NR |
| Outcome data | 15* | *Cohort study*—Report numbers of outcome events or summary measures over time | NR | NR |
|  |  | *Case-control study—*Report numbers in each exposure category, or summary measures of exposure | NR | NR |
|  |  | *Cross-sectional study—*Report numbers of outcome events or summary measures | *9* | *Table 1* |
| Main results | 16 | (*a*) Give unadjusted estimates and, if applicable, confounder-adjusted estimates and their precision (eg, 95% confidence interval). Make clear which confounders were adjusted for and why they were included | 19 | Table 4 |
|  |  | (*b*) Report category boundaries when continuous variables were categorized | 19 | Table 4 |
|  |  | (*c*) If relevant, consider translating estimates of relative risk into absolute risk for a meaningful time period | NR | NR |

Continued on next page

| Other analyses | 17 | Report other analyses done—eg analyses of subgroups and interactions, and sensitivity analyses | ND | ND |
| --- | --- | --- | --- | --- |
| Discussion | | | | |
| Key results | 18 | Summarise key results with reference to study objectives | 20 | Overall, our study highlights three major findings |
| Limitations | 19 | Discuss limitations of the study, taking into account sources of potential bias or imprecision. Discuss both direction and magnitude of any potential bias | 21 | Although our study did not address genetic susceptibility to dermatophytosis, |
| Interpretation | 20 | Give a cautious overall interpretation of results considering objectives, limitations, multiplicity of analyses, results from similar studies, and other relevant evidence | 21 | These differences between crude and adjusted risk estimates in this study are caused by multicollinearity, nested effects (i.e. contact with animals and contact with dogs … ) or non-independence with quasi-complete separation of data points (i.e. type of hairdressing mode (braiding or head shaving) according to the gender) among the predictors in the multivariate analysis |
| Generalisability | 21 | Discuss the generalisability (external validity) of the study results | 20 | the schoolchildren surveyed in each area were homogenous, especially in regards to age, and each survey was performed at the same period during the dry season |
| Other information | |  | | |
| Funding | 22 | Give the source of funding and the role of the funders for the present study and, if applicable, for the original study on which the present article is based | - | Detailed in the electronic submission form. |

*Give information separately for cases and controls in case-control studies and, if applicable, for exposed and unexposed groups in cohort and cross-sectional studies.

**Note:** An Explanation and Elaboration article discusses each checklist item and gives methodological background and published examples of transparent reporting. The STROBE checklist is best used in conjunction with this article (freely available on the Web sites of PLoS Medicine at http://www.plosmedicine.org/, Annals of Internal Medicine at http://www.annals.org/, and Epidemiology at http://www.epidem.com/). Information on the STROBE Initiative is available at www.strobe-statement.org.
