# Supplementary material for: Dermatophytosis among Schoolchildren in Three Eco-climatic Zones of Mali
Source: PLoS Negl Trop Dis. 2016 Apr 28;10(4):e0004675. doi: 10.1371/journal.pntd.0004675 (PMC4849727; doi:10.1371/journal.pntd.0004675)
Supplement: S1 Table — (DOCX) [file pntd.0004675.s002.docx]

**Supplementary Table 1. Common hairdressing habits and domestic animal contact among the 590 children, according to gender and *tinea capitis* status.**

|  | **Study population** | | | | | | |  |  | **Females (n=304)** | |  | |  | | |  | | **Males (n=286)** | | | | |  |  |
| --- | --- | --- | --- | --- | --- | --- | --- | --- | --- | --- | --- | --- | --- | --- | --- | --- | --- | --- | --- | --- | --- | --- | --- | --- | --- |
|  | **Overall (n)** | | |  | ***Tinea capitis*** | | | P **value** |  | ***Tinea capitis*** | | *P* **value** | | |  | | ***Tinea capitis*** | | | | | | |  | |
| **Risk factors** | **n=590** | | **%** |  | **n=232** | | **%** |  |  | **n=90** | **%** |  | | |  | | **n=142** | | | | **%** | | ***P* value** | | |
| Public hairdressing | 253 | 42.9 | |  | 174 | 75 | | 0.022 |  | 4 | 4.4 | 0.490 | | |  | | 125 | | | 88.0 | | 0.186 | | | |
| Home hairdressing | 342 | 57.9 | |  | 129 | 55.6 | | <0.001 |  | 89 | 98.9 | 0.678 | | |  | | 18 | | | 12.7 | | 0.194 | | | |
| Traditional braiding | 303 | 51.4 | |  | 107 | 46.1 | | <0.001 |  | 90 | 100 | 0.558 | | |  | | 1 | | | 0.7 | | 1.000 | | | |
| Head shaving | 286 | 48.4 | |  | 141 | 60.8 | | <0.001 |  | 0 | 0 | 1.000 | | |  | | 141 | | | 99.3 | | 1.000 | | | |
| Hairdressing frequency, Mean (SD) | 1.7 | (0.9) | |  | 1.7 | (0.9) | | 0.531 |  | 1.8 | (0.9) | | 0.430 | | |  | | 1.7 | | (0.9) | | 0.408 | | | |
| Nb children sleeping /room, Mean (SD) | 3.3 | (1.7) | |  | 3.3 | (1.7) | | 0.847 |  | 3.4 | (1.5) | | 0.226 | | |  | | 3.3 | | (1.9) | | 0.248 | | | |
| Domestic animals | 540 | 91.5 | |  | 212 | 91.4 | | 0.918 |  | 76 | 84.4 | | 0.064 | | | |  | | 136 | 95.8 | | 0.223 | | | |
| Contact with animals | 409 | 69.4 | |  | 174 | 75.0 | | 0.022 |  | 58 | 64.4 | | 0.608 | | | |  | | 116 | 81.7 | | 0.090 | | | |
| Dog | 227 | 38.5 | |  | 107 | 46.1 | | 0.002 |  | 36 | 40.0 | | 0.293 | | | |  | | 71 | 50.0 | | 0.008 | | | |
| Cat | 180 | 30.5 | |  | 77 | 33.2 | | 0.273 |  | 27 | 30.0 | | 1.000 | | | |  | | 50 | 35.2 | | 0.095 | | | |
| Horse | 41 | 7.0 | |  | 11 | 4.7 | | 0.099 |  | 5 | 5.6 | | 0.630 | | | |  | | 6 | 4.2 | | 0.153 | | | |
| Sheep | 344 | 58.3 | |  | 127 | 54.7 | | 0.176 |  | 43 | 47.8 | | 0.058 | | | |  | | 84 | 59.2 | | 0.717 | | | |
| Goat | 158 | 26.8 | |  | 64 | 27.6 | | 0.775 |  | 26 | 28.9 | | 0.890 | | | |  | | 38 | 26.8 | | 0.587 | | | |
| Cow | 178 | 30.17 | |  | 78 | 33.6 | | 0.143 |  | 27 | 30.0 | | 0.678 | | | |  | | 51 | 35.9 | | 0.206 | | | |
| Donkey | 264 | 44.8 | |  | 113 | 48.7 | | 0.128 |  | 39 | 43.3 | | 0.612 | | | |  | | 74 | 52.1 | | 0.287 | | | |
| Poultry | 481 | 81.5 | |  | 192 | 82.8 | | 0.588 |  | 70 | 77.8 | | 0.759 | | | |  | | 122 | 85.9 | | 0.517 | | | |
| Proximity to cattle | 349 | 59.2 | |  | 124 | 53.5 | | 0.026 |  | 44 | 48.9 | | 0.043 | | | |  | | 80 | 56.3 | | 0.183 | | | |
